# Mental Health Professionals’ attitudes towards trans people

**DOI:** 10.1192/j.eurpsy.2022.2076

**Published:** 2022-09-01

**Authors:** M.A. Cutillas Fernández, J.A. Jiménez Barbero

**Affiliations:** 1 Hospital Morales Meseguer, Psiquiatría, Murcia, Spain; 2 Universidad de Murcia, Enfermería, Murcia, Spain

**Keywords:** trans mental health, attitudes, trans

## Abstract

**Introduction:**

Since the emergence of the term “transsexualism” in the Ninth International Classification of Diseases (ICD-9), disciplines related to mental health have contributed to the perpetuation of stereotypical attitudes towards trans people. Recent years have shown the significant prevalence of mental pathology suffered by this group, and the need for specialized training to improve access to the health system.

**Objectives:**

The main objectives of this research are: (a) to find and analyse the scientific evidence published which assesses the attitudes of mental health professionals towards the trans community; b) to establish the main variables that modify these attitudes, paying special attention to gender, ideology, sexual orientation and previous training or experience

**Methods:**

A systematic review of the literature was conducted following the PRISMA recommendations.

**Results:**

Tendency towards more positive and liberal attitudes among professionals than in the general population.

Higher values for extreme prejudice among those professionals who attributed gender diversity to a psychological, ethico-moral or religious cause.

Association of depathologising practices with belief in the psychosocial nature of diversity, clinical training and interpersonal contact with LGBT people. The following socio-demographic variables were related: being a woman, clinical psychologists, progressive political ideology, professionals who strive to know their own limitations and biases.

**Conclusions:**

More positive attitudes than the general population but still insufficient Specific training in gender diversity and minority issues would be a key element in improving care for transgender people. The attitudes of professionals depend, in part, on the personal characteristics of the therapists.

**Disclosure:**

No significant relationships.

